# Adjuvant chemo-radiation for gastric adenocarcinoma: an institutional experience

**DOI:** 10.1186/1748-717X-5-50

**Published:** 2010-06-04

**Authors:** Philippe G Aftimos, Elie A Nasr, Dolly I Nasr, Roger J Noun, Fady L Nasr, Marwan G Ghosn, Joelle A El Helou, Georges Y Chahine

**Affiliations:** 1 Hematology - Oncology Department, Hotel Dieu de France University Hospital, Alfred Naccache BLVD, Achrafieh, Beirut, Lebanon; 2 Radiation Oncology Department, Hotel Dieu de France University Hospital, Alfred Naccache BLVD, Achrafieh, Beirut, Lebanon; 3 General Surgery Department, Hotel Dieu de France University Hospital, Alfred Naccache BLVD, Achrafieh, Beirut, Lebanon

## Abstract

**Background:**

Studies have shown that surgery alone is less than satisfactory in the management of early gastric cancer, with cure rates approaching 40%. The role of adjuvant therapy was indefinite until three large, randomized controlled trials showed the survival benefit of adjuvant therapy over surgery alone. Chemoradiation therapy has been criticized for its high toxicity.

**Methods:**

24 patients diagnosed between September 2001 and July 2007 were treated with adjuvant chemoradiation. 18 patients had the classical MacDonald regimen of 4500 cGy of XRT and chemotherapy with 5-fluorouracil (5FU) and leucovorin, while chemotherapy consisted of 5FU/Cisplatin for 6 patients.

**Results:**

This series consisted of non-metastatic patients, 17 females and 7 males with a median age of 62.5 years. 23 patients (96%) had a performance status of 0 or 1. The full course of radiation therapy (4500 cGy) was completed by 22 patients (91.7%). Only 7 patients (36.8%) completed the total planned courses of chemotherapy. 2 local relapses (10%), 2 regional relapses (10%) and 2 distant relapses (10%) were recorded. Time to progression has not been reached. 9 patients (37.5%) died during follow-up with a median overall survival of 75 months. Patients lost a mean of 4 Kgs during radiation therapy. We recorded 6 episodes of febrile neutropenia and the most frequent toxicity was gastro-intestinal in 17 patients (70.8%) with 9 (36%) patients suffering grade 3 or 4 toxicity and 5 patients (20%) suffering from grade 3 or 4 neutropenia. 4 (17%) patients required total parenteral nutrition for a mean duration of 20 days. 4 patients suffered septic shock (17%) and 1 patient developed a deep venous thrombosis and a pulmonary embolus.

**Conclusions:**

Adjuvant chemo-radiation for gastric cancer is a standard at our institution and has resulted in few relapses and an interesting median survival. Toxicity rates were serious and this remains a harsh regimen with only 36.8% of patients completing the full planned courses of chemotherapy. This is due to hematological toxicity, mainly febrile neutropenia. This should prompt us to review the subsequent chemotherapy protocol and make it more tolerable.

## Background

While gastric cancer incidence is second only to lung cancer worldwide, with an estimated 870,000 new cases and 650,000 deaths every year and the high-risk areas being East Asia, South America, and Eastern Europe [1,2], the age standardized incidence rate in Lebanon between 1998 and 2002 is 7 per 100 000 for men and 4.6 per 100 000 for women [2]. Gastric cancer constituted 5.1% of all cancer cases in Lebanon in the first national population-based registry in 1998 [3]. The curative treatment of gastric cancer requires surgical resection [4]. Nevertheless, studies have shown that surgery alone is less than satisfactory with cure rates approaching 40% [5]. Worldwide, large amounts of resources have been expended in the search for an effective adjuvant therapy to reduce the risk of relapse following surgery for gastric cancer. After several decades of investigation that have yielded little improvement in survival rates, three large, randomized controlled trials showed the survival benefit of adjuvant therapy over surgery alone. In 2001, the American INT 0116 demonstrated that chemoradiotherapy after resection for gastric cancer significantly improves relapse-free and overall survival [6]. Adjuvant chemoradiotherapy has been adopted since as standard of care in the USA. Nevertheless, it is still uncommonly used in other countries such as Britain. This relates mainly to criticism of the INT0116 trial with regards to suboptimal surgery, toxicity, an outdated chemotherapy regimen and suboptimal radiotherapy techniques. Subsequently, the final results of The Medical Research Council MAGIC study on perioperative chemotherapy [7] provoked thoughtful discussion regarding the relative efficacy of neoadjuvant chemotherapy compared with INT0116 chemoradiation after surgery. Both studies assessed the benefits of adjuvant therapy after only limited surgery. The Adjuvant Chemotherapy Trial of TS-1 for Gastric Cancer (ACTS-GC) showed a survival benefit of S1 adjuvant monotherapy after curative D2 surgery in patients with stage II or III gastric cancer [8]. Several investigations have been made regarding the optimal chemotherapy regimen in order to improve treatment efficacy and reduce its toxicity. Moreover, the wide availability of 3-dimensional treatment planning systems and the technological developments in the delivery of radiation therapy, promises improvements in the therapeutic ratio as well as a potential to reduce treatment-related toxicity. This paper reports the results and toxicity profile of adjuvant chemoradiation experience at the radiation oncology department of Hotel-Dieu de France University Hospital in Beirut, Lebanon.

## Methods

This is a retrospective analysis of a series of patients recruited from the database of the radiation oncology department of Hotel-Dieu de France University Hospital in Beirut Lebanon. Between September 2001 and July 2007, 24 patients with pathologically confirmed adenocarcinoma of the gastro-oesophageal junction or the stomach underwent surgery with curative intent and were treated with adjuvant chemoradiation. These patients were treated and included in the database only if they met the following criteria. All the patients had their tumor staged according to the 6^th ^edition of the American Joint Commission on Cancer (AJCC) Cancer Staging Manual, and metastatic patient were excluded. An Eastern Cooperative Oncology Group (ECOG) performance status had been recorded for all the patients. The eligibility criteria for treatment included a serum creatinine (mg/dL) ≤ 1.5, total bilirubin ≤ 1.5 mg/dL, and alanine aminotransferase (ALT) ≤ 1.5 × upper limit of normal. Patients in the study did not receive preoperative chemotherapy. Treatment was begun as soon as possible and not later than 8 weeks after surgery. The pretreatment evaluations included physical examination, tumor markers and computed tomography (CT) to rule out metastatic disease.

18 patients received from the classical MacDonald regimen of radiation therapy (XRT) and chemotherapy with 5-fluorouracil and leucovorin (5FU/LV), while chemotherapy consisted of 5FU/Cisplatin for 6 patients for the same duration of treatment (5FU: 800 mg/m2 d1-d5/cisplatin 75 mg/m2 d2).

Radiotherapy was delivered following the recommendations outlined in INT0116 [6].

All patients were treated using a standardized 3D conformal technique. When available, the preoperative and postoperative scans were reviewed and endoscopy, surgical, and pathology reports were also reviewed. Patients had a CT simulation performed at least 1 week before starting radiotherapy. The CT simulation slice thickness was 5 mm. Patients were scanned in the supine position with arms above their head. A total radiation dose of 45 Gy was delivered in 25 fractions at 1.8 Gy per fraction, five days per week over five weeks. Dose variation in the planning target volume (PTV) was kept within +7 and -5% of the prescribed dose in accordance with ICRU 50/62 recommendations. Radiation was delivered using 6-18 MV photons with a linear accelerator. The clinical target volume (CTV) and the design of the radiation treatment fields were individualized depending upon the extent and location of the primary tumor and involved lymph nodes, and the type of surgery performed. Lymph node stations in the radiation fields included perigastric, coeliac, splenic hilar, suprapancreatic, porta hepatis, pancreaticoduodenal and local paraaortic nodes. In patients with tumors of the gastroesophageal junction, paracardial and paraesophageal lymph nodes were included in the radiation fields, but pancreaticoduodenal radiation was not required. The planning target volume consisted of the CTV with a 1cm margin. The organs at risk were contoured, which included kidneys, liver, heart, and spinal cord. At least two third of one kidney was spared. Not more than 50% of the heart received more than 40 Gy and not more than 70% of the liver received more than 30 Gy. The maximum spinal cord dose was less than 45 Gy. The dose-volume histogram was used to ensure that the dose tolerances were met for the nearby critical organs.

Acute toxicity data were graded according to the RTOG Acute Radiation Morbidity Scoring Criteria [9], while hematologic toxicity was graded using NCI-CTC version 3. Postoperative follow-up by the treating oncologist was scheduled every 4 months for the first 2 years and every 6 months after 2 years. Follow-up included detailed history taking and physical examination. No routine endoscopy nor CT scans (chest, abdomen, pelvis) were done unless clinically warranted while patients were followed with routine CBC, chemistry and CA 19-9 at every follow-up (as per GAST-5 NCCN guidelines). Sites of first failure (ie, locoregional or distant) were also collected.

Locoregional recurrence was defined as any recurrence in the tumor bed, anastomosis site, gastric remnant, duodenal stump, and regional nodes within the irradiated volume.

Distant metastases were defined as any recurrence outside of the irradiated field, including metastases to the liver, lower para-aortic lymph nodes, and extra-abdominal sites and peritoneal seeding. Time to progression was measured from the date of radical surgery to the date of first recurrence of disease. Overall survival was also recorded from the date of radical surgery till death from any cause.

The data were analyzed using SPSS version 11.0. Patient survival was calculated using the method of Kaplan-Meier.

## Results

The cohort consisted of 24 patients, 17 males and 7 females. Patient and tumor characteristics are outlined in Tables 1. The mean age was 58.3 years (range, 35-80) and median age was 62.5. 23 patients (96%) had a performance status (PS) of 0 or 1. Thirteen (54%) underwent subtotal gastrectomy and 11 had total gastrectomy (46%). Twenty-three patients (96%) had negative margins. One patient had infiltrated surgical margins. Stage of disease was IB in 2 patients (8.3%), II in 9 patients (37.5%), IIIA in 8 patients (33.3%), IIIB in 4 patients (16.4%) and IV (no metastasis) in 1 patient (4.2%). The majority of patients (75%) had T3 primary tumors. Seventeen patients (71%) had regional nodal involvement. 21 patients had a D1 nodal clearance and 3 patients had D2 surgery.

**Table 1:** Patient and tumor characteristics

characteristics	N (%)
**Total**	
Males	17(71%)
Females	7(29%)
**Age**	
Mean	58.3
< 50	6(25%)
50-69	16 (67%)
> 70	2(8%)
**PS**	
0	18(75%)
1	5(21%)
2	1(4%)
**Pathologic tumor stage**	
T1	1(4%)
T2	5(21%)
T3	18(75%)
T4	0
**Pathologic lymph node status**	
N0	7(29%)
N1	11(46%)
N2	5(21%)
N3	1(4%)

The median follow-up was 11.5 months and mean follow-up 21.2 months. 6 patients were lost to follow-up.

The full course of radiation therapy (4500 cGy) was completed by 22 patients (91.7%) with 1 patient continuing it at a foreign institution and 1 patient interrupting it for grade IV gastro-intestinal toxicity. Only 7 patients (36.8%) completed the total planned courses of chemotherapy, 4 patients that received 5FU/LV and 3 patients that received 5FU/cisplatin. Acute toxicity was recorded during the concurrent chemoradiation regimen and for the entire adjuvant chemotherapy cycles. It is described in table 2. The most common severe acute adverse effect was gastrointestinal in 17 patients (70.8%) with 36% grade 3 or 4 toxicity. Mucositis came in second with 32% grade 3 or 4 toxicity. Hematologic toxicity was the third major toxicity with 20% of patients developing grade 3 or 4 neutropenia and 16% of patients suffering from grade 3 or 4 anemia. 6 episodes of febrile neutropenia were recorded. Patients lost a mean of 7% of body weight during radiation therapy. 25% of patients lost 10% or more of body weight while 45% of patients lost between 5 and 10% of their initial body weight during radiation therapy. 4 (17%) patients required total parenteral nutrition for a mean duration of 20 days (range 3-60 days). 4 patients suffered septic shock (17%) and 1 patient developed a deep venous thrombosis and a pulmonary embolus. Toxicity pattern in patients receiving 5FU and cisplatin (n = 6) was comparable to the entire cohort: 50% grade 3 or 4 gastrointestinal toxicity, 33% grade 3 or 4 mucositis, 33% grade 3 or 4 neutropenia, 33% grade 3 or 4 anemia.

**Table 2:** Acute toxicity

	Grade 1		Grade 2		Grade 3		Grade 4		Grade 3 & 4	
**Adverse Events**	**n**	**%**	**n**	**%**	**n**	**%**	**n**	**%**	**n**	**%**

Mucositis	0	0.0	0	0.0	3	12.0	5	20.0	8	32.0
GI	3	12.0	5	20.0	2	8.0	7	28.0	9	36.0
Neutropenia	3	12.0	3	12.0	1	4.0	4	16.0	5	20.0
Anemia	5	20.0	5	20.0	4	16.0	0	0.0	4	16.0
Thrombocytopenia	4	16.0	1	4.0	1	4.0	0	0.0	1	4.0
Dermatitis	0	0.0	0	0.0	1	4.0	0	0.0	1	4.0

Among 24 patients in the entire cohort, 4 patients (22%) relapsed with disease during the follow-up period. The patterns of failure are described in table 3. Two patients had an isolated locoregional recurrence, and 2 relapsed with both locoregional and distant disease. All of the patients who relapsed had negative pathologic margins. The median time to relapse from the end of the radiation was 10.5 months (range 5-18).

**Table 3:** Patterns of failure

	N	%
**Local Relapse (N = 18)**		
Yes	2	11.0
No	16	89.0
**Regional Relapse (N = 18)**		
Yes	2	11.0
No	16	89.0
**Distant Relapse (N = 18)**		
Yes	2	11.0
No	16	89.0

**Figure 1:**
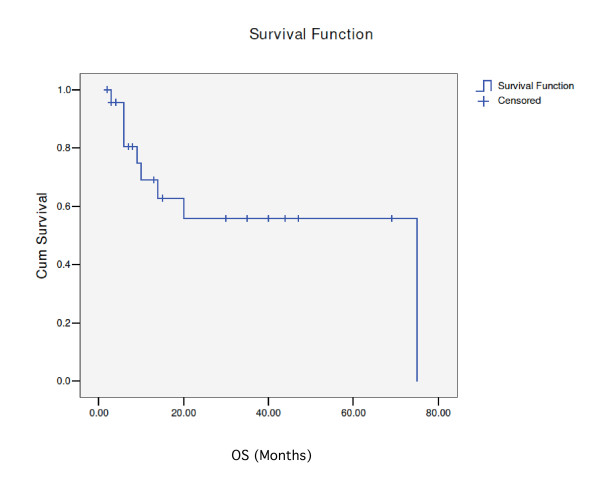
**overall survival curve**.

**Figure 2:**
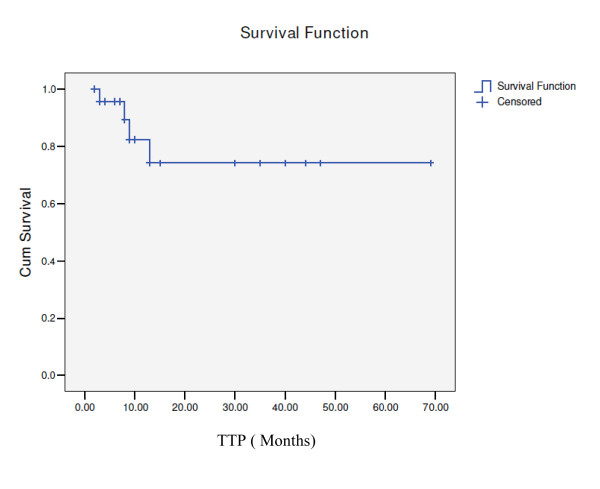
**time to progression curve**.

18 patients were followed-up for survival. In the entire cohort, the median survival was 75 months while time to progression has not been reached (Figs.1 and 2). 9 patients (37.5%) died during follow-up. All 4 patients that experienced relapse died. 5 patients died in remission, 4 of which were considered toxic deaths: one with a massive pulmonary embolus, one from extreme cachexia and three from septic shock.

## Discussion

The five-year survival rate for patients with completely resected early gastric cancer is approximately 75 percent [10], while it is 30 percent or less for patients who have extensive lymph node involvement [11]. A Dutch randomized trial published in 1999 did not support the routine use of D2 over D1 lymphadenectomy in terms of survival [12]. These sobering results have spawned efforts to improve the treatment results for this group of patients using adjuvant or neoadjuvant radiation therapy and/or chemotherapy.

The major risk factors for stomach cancer are hypothesized to be nutritional [13]. The Middle East has good access to fruits and vegetables throughout the year, and the nutritional practices have Mediterranean influences. This may result in the low incidence rates that were observed. However, smoking carries a slightly increased risk for stomach cancer [14] and smoking is quite common in countries of the Middle East.

The favored adjuvant strategy at our institution is the Intergroup trial 0116 chemoradiotherapy regimen. Our 7 years experience seems commensurate with that of INT-0116. The treated population has similar characteristics: median age was 62.5 years old comparable to 60 in the chemoradiotherapy group of INT-0116. Stomach cancer is among those cancers that are more difficult to detect, and is mostly diagnosed at a later stage. 75% of patients in our series had T3 tumors, as were the majority in INT-0116 with 68 and 69% of T3-T4 in the treated and control groups respectively. Nodal status demonstrates advanced disease with 85% positive in INT-0116. 70% of patients in this described series had positive nodes. Our results however are incomparable for the main reason that this series is retrospective and descriptive of only 24 patients while INT-0116 is a prospective randomized study of 556 patients. Second, the follow-up period and the little number of events only allowed a median survival analysis while time to progression has not yet been reached. This retrospective work has helped us evaluate our chosen institutional adjuvant gastric cancer strategy in terms of relapse and survival. We have specially learned from the criticism of the American Intergroup trial about the limited extent of the surgical procedure in many cases. Being a multi-centre trial with the lack of monitoring of the quality of surgery, D2 lymph node dissection was only performed in 10% of the enrollees, and 54% did not even have clearance of the D1 nodal regions. Gastric oncologic surgery at our institution is performed by one academic surgeon and negative gastrectomy margins with D1 nodal dissection or more have been implemented as a requirement for enrollment in the chemoradiotherapy regimen. All patients in the described series had therefore benefited from D1 or D2 resection.

However, toxicity remains a problem and this regimen is harsh on patients. Historically peri-operative morbidity and mortality are 25% and 4% respectively for D1 resection, and 43% and 10% respectively for D2 resection [4]. 36% of patients of the chemoradiotherapy arm of INT-0116 did not complete the regimen while grade 3 and grade 4 toxic effects occurred in 41 and 32% of cases respectively. While most of our patients (91.7%) managed to go through the full radiation therapy course, more than half (63.2%) didn't complete the whole treatment plan with a mean weight loss of 4 kilograms between the beginning and end of treatment. Even though worse and most frequent adverse events were, as in INT-0116, gastro-intestinal and hematological, the rate of serious toxicity in this described series is high. 4 toxic deaths have been counted (17%) compared to only 1% in INT-0116.The numbers of grade 3 or 4 neutropenia (20%) and septic shocks (17%) need to be considered. This should prompt us to evaluate other adjuvant strategies, the MAGIC regimen notably [7] or other treatment delivery strategies: postoperative chemoradiation in combination with preoperative chemotherapy (CRITICS trial: Clinicaltrials.gov NCT 00407186), the role of adding bevacizumab to perioperative chemotherapy (MAGIC-B study: MRC-ST03).

## Conclusions

The results of adjuvant chemoradiotherapy have changed the standard of care in the US following potentially curative resection of gastric cancer. Even more, support for the benefit of adjuvant chemoradiotherapy in patients who have undergone a therapeutic D2 lymph node dissection was provided by a retrospective review of 990 Korean patients whose 5-year overall survival and relapse-free survival rates significantly favor chemoradiotherapy [15]. The optimal regimen for postoperative chemoradiotherapy has not yet been established. The future research is on optimizing the chemotherapy regimen, defining the role of radiotherapy, it's integration in the treatment schema (pre or postoperative) and exploring the effect of treatment timing (preoperative, postoperative or both). Moreover, targeted therapies have recently made their way in gastric cancer with the approval of trastuzumab for the treatment of HER-2 positive metastatic gastric cancer [16] Anti-angiogenics (bevacizumab) and anti-EGFR agents (cetuximab) are also being studied in the adjuvant setting. Future questions arise on the role of these therapies in the adjuvant setting. Would their inclusion in adjuvant chemotherapy regimens surpass the need for radiation therapy?

## Competing interests

The authors declare that they have no competing interests.

## Authors' contributions

PA conceived the study, reviewed all patient files and drafted the manuscript. EN participated in the design amd coordination of the study, and corrected the manuscript. DN offered help in writing the radiation techniques part. RN offered access to patient files and provided help with the surgery technique part. FN and MG offered access to their patients' files. JEH drafted the material and methods part of the manuscript. GC participated in the design of the study, offered resources for the statistical analysis and provided access to his patients' files. All authors read and approved the final manuscript.

## Authors' information

GC is chairman of the hematology and medical oncology department at Hotel-Dieu de France University Hospital.

EN is chairman of the radiation oncology department at Hotel-Dieu de France University Hospital.

RN is the gastric surgery specialist at Hotel-Dieu de France University Hospital.

## References

[B1] Parkin DM Pisani P Ferlay J Estimates of the worldwide incidence of 25 major cancers in 1990 Int J Cancer 1999 80 827 41 10.1002/(SICI)1097-0215(19990315)80:6<827::AID-IJC6>3.0.CO;2-P 10074914

[B2] Ferlay J Bray F Pisani P Parkin DM GLOBOCAN 2002: cancer incidence. Mortality and prevalence worldwide IARC cancer base no. 5, version 2.0 2004 Lyon (France): IARC Press

[B3] Shamseddine A Sibai AM Gehchan N Rahal B El-Saghir N Ghosn M Aftimos G Chamsuddine M Seoud M For The Lebanese Cancer Epidemiology Group: Cancer incidence in postwar Lebanon: findings from the first national population-based registry, 1998 Ann Epidemiol 2004 14 663 668 10.1016/j.annepidem.2003.12.002 15380797

[B4] Dupont JB Jr Lee JR Burton GR Cohn I Jr Adenocarcinoma of the stomach: review of 1,497 cases Cancer 1978 41 941 947 10.1002/1097-0142(197803)41:3<941::AID-CNCR2820410323>3.0.CO;2-M 638980

[B5] Hundahl SA Phillips JL Menck HR The National Cancer Data Base fifth edition American Joint Committee on Cancer staging proximal disease, and the "different disease" hypothesis Cancer 2000 88 921 932 10.1002/(SICI)1097-0142(20000215)88:4<921::AID-CNCR24>3.0.CO;2-S 10679663

[B6] Macdonald JS Smalley SR Benedetti J Hundahl SA Estes NC Stemmermann GN Haller DG Ajani JA Gunderson LL Jessup JM Martenson JA Chemoradiotherapy after surgery compared with surgery alone for adenocarcinoma of the stomach or gastroesophageal junction N Engl J Med 2001 345 725 730 10.1056/NEJMoa010187 11547741

[B7] Cunningham D Allum WH Stenning SP Thompson JN Van de Velde CJ Nicolson M Scarffe JH Lofts FJ Falk SJ Iveson TJ Smith DB Langley RE Verma M Weeden S Chua YJ MAGIC Trial Participants: Perioperative chemotherapy versus surgery alone for resectable gastroesophageal cancer N Engl J Med 2006 355 11 20 10.1056/NEJMoa055531 16822992

[B8] Sakuramoto S Sasako M Yamaguchi T Kinoshita T Fujii M Nashimoto A Furukawa H Nakajima T Ohashi Y Imamura H Higashino M Yamamura Y Kurita A Arai K ACTS-GC Group: Adjuvant chemotherapy for gastric cancer with S-1, an oral fluoropyrimidine N Engl J Med 2007 357 1810 1820 10.1056/NEJMoa072252 17978289

[B9] Radiation Therapy Oncology Group (RTOG) Acute Radiation Morbidity Scoring Criteria http://www.rtog.org/members/toxicity/acute.html

[B10] Middleton G Cunningham D Current options in the management of gastrointestinal cancer Ann Oncol 1995 6 Suppl 1 17 25 discussion 25-6 8695539 10.1093/annonc/6.suppl_1.s17

[B11] Agboola O Adjuvant treatment in gastric cancer Cancer Treat Rev 1994 20 217 240 10.1016/0305-7372(94)90001-9 8020004

[B12] Bonenkamp JJ Hermans J Sasako M Van de Velde CJ Welvaart K Songun I Meyer S Plukker JT Van Elk P Obertop H Gouma DJ van Lanschot JJ Taat CW de Graaf PW von Meyenfeldt MF Tilanus H Dutch Gastric Cancer Group: Extended lymph-node dissection for gastric cancer N Engl J Med 1999 340 908 914 10.1056/NEJM199903253401202 10089184

[B13] World Cancer Research Fund and American Institute for Cancer Research. Food nutrition and the prevention of cancer: a global perspective 1997 Washington(DC): World Cancer Research Fund 10.1016/s0899-9007(99)00021-0 10378216

[B14] Koizumi Y Tsubono Y Nakaya N Kuriyama S Shibuya D Matsuoka H Tsuji I Cigarette smoking and the risk of gastric cancer: a pooled analysis of two prospective studies in Japan Int J Cancer 2004 112 1049 1055 10.1002/ijc.20518 15386347

[B15] Kim S Lim DH Lee J Kang WK MacDonald JS Park CH Park SH Lee SH Kim K Park JO Kim WS Jung CW Park YS Im YH Sohn TS Noh JH Heo JS Kim YI Park CK Park K An observational study suggesting clinical benefit for adjuvant postoperative chemoradiation in a population of over 500 cases after gastric resection with D2 nodal dissection for adenocarcinoma of the stomach Int J Radiat Oncol Biol Phys 2005 63 1279 1285 10.1016/j.ijrobp.2005.05.005 16099596

[B16] Van Cutsem E Kang Y Chung H Shen L Sawaki A Lordick F Hill J Lehle M Feyereislova A Banget Y Efficacy results from the ToGA trial: A phase III study of trastuzumab added to standard chemotherapy (CT) in first-line human epidermal growth factor receptor 2 (HER2)- positive advanced gastric cancer (GC) J Clin Oncol 2009 27 18s (suppl; abstr LBA4509) 10.1200/JCO.2009.22.4626

